# Recomendaciones sobre vacunación en niños y adolescentes con errores innatos de la inmunidad según el programa ampliado de inmunización colombiano

**DOI:** 10.7705/biomedica.7424

**Published:** 2024-12-23

**Authors:** Nathalia Cortés-Marín, Luis Miguel Sosa-Ávila, Andrés Felipe Arias, Leonardo David Escobar-Cortés, Juan Pablo Rojas-Hernández

**Affiliations:** 1 Departamento de Pediatría, Hospital Universitario del Valle, Cali, Colombia Hospital Universitario del Valle Departamento de Pediatría Hospital Universitario del Valle Cali Colombia; 2 Departamento de Pediatría, Universidad Industrial de Santander, Bucaramanga, Colombia Universidad Industrial de Santander Departamento de Pediatría Universidad Industrial de Santander Bucaramanga Colombia; 3 Departamento de Pediatría, Universidad de Santander, Cúcuta, Colombia Universidad de Santander Universidad de Santander Cúcuta Colombia; 4 Departamento de Enfermedades Infecciosas, IPS de la Costa, Barranquea, Colombia IPS de la Costa Departamento de Enfermedades Infecciosas IPS de la Costa Barranquea Colombia; 5 Departamento de Pediatría, Universidad Libre - Seccional Cali, Cali, Colombia Universidad Libre Departamento de Pediatría Universidad Libre Seccional Cali Cali Colombia; 6 Departamento de Pediatría, Universidad del Valle, Cali, Colombia Universidad del Valle Departamento de Pediatría Universidad del Valle Cali Colombia; 7 Departamento de Pediatría, Pontificia Universidad Javeriana, Cali, Colombia Pontificia Universidad Javeriana Departamento de Pediatría Pontificia Universidad Javeriana Cali Colombia; 8 Departamento de Pediatría, Universidad San Martín de Cali, Cali, Colombia Universidad San Martín de Cali Departamento de Pediatría Universidad San Martín de Cali Cali Colombia

**Keywords:** inmunidad, vacunación, inmunización, niño, adolescente., Immunity, vaccination, immunization, child, adolescent.

## Abstract

En el presente manuscrito se presenta un análisis exhaustivo de las recomendaciones mundiales sobre inmunización en pacientes con errores innatos de la inmunidad. Se examinaron los mecanismos de acción y los tipos de vacunas, y se describieron las vacunas incluidas en el Plan Ampliado de Inmunización (PAI) colombiano y las pautas específicas para la inmunización de pacientes con los errores innatos de la inmunidad más frecuentes en Colombia.

Estas recomendaciones se ajustaron según la gravedad y la subclasificación de cada inmunodeficiencia, teniendo en cuenta las variaciones en la respuesta inmunitaria, con el objetivo de ofrecer recomendaciones basadas en la evidencia clínica para la vacunación de niños con estas condiciones. Se contemplaron los errores de la inmunidad más comunes a nivel global y las vacunas incluidas en el PAI colombiano, para evitar retrasos en los esquemas de vacunación.

Todo esto se logró mediante una revisión narrativa, no sistemática, de artículos indexados en español y en inglés, buscados con los términos MeSH: “errores innatos de la inmunidad”, “inmunodeficiencias primarias”, “vacunación en errores innatos de la inmunidad”, “tipos de vacunas”, “mecanismo de acción de las vacunas” y “vacunas vivas en errores innatos de la inmunidad”.

Se emplearon motores de búsqueda como: PubMed, Medline, ScienceDirect y páginas web de instituciones reconocidas como *Centers for Disease Control and Prevention* (CDC).

## Abstract (en)

In this manuscript, we carried out an exhaustive analysis of the global recommendations for immunization in inborn errors of immunity patients. We examined the mechanisms of action and types of vaccines, and we described the vaccines included in the Colombian immunization program together with the specific guidelines for immunization in patients with the most frequent inborn errors of immunity in Colombia.

These recommendations were adjusted according to the severity and subclassifications of each immunodeficiency, considering variations in the immune response to offer evidence- based recommendations for vaccination in children with these conditions. We included the most common inborn errors of immunity worldwide and considered the vaccines included in the Colombian immunization program to avoid delays in vaccination schedules.

This work was achieved through a narrative, non-systematic review of articles indexed in Spanish and English, using MeSH terms such as: “inborn errors of immunity”, “primary immunodeficiencies”, “vaccination in inborn errors of immunity, “types of vaccines”, “mechanism of action of vaccines”, and “live vaccines in inborn errors of immunity”.

We used search engines such as: PubMed, Medline, ScienceDirect, and websites of recognized institutions such as the Centers for Disease Control and Prevention (CDC).

La vacunación es la medida más efectiva en salud pública a nivel mundial, ya que previene billones de muertes alrededor del mundo ^1^^,^^2^ y discapacidades a largo plazo, además de ser segura y asequible ^3^. Por estas razones, se ha convertido en un pilar fundamental para la protección de la salud de la población infantil y adolescente en Colombia. Sin embargo, es esencial reconocer que existen condiciones médicas especiales, como los errores innatos de la inmunidad, que requieren de atención personalizada en lo que respecta a la inmunización ^4^.

Los errores innatos de la inmunidad, previamente denominados inmunodeficiencias primarias, representan un grupo de 450 enfermedades, aproximadamente, que se caracterizan por alteraciones cuantitativas o funcionales del sistema inmunológico ^5^^,^^6^, lo que genera mayor propensión a infecciones -usualmente más graves y recurrentes-y con una mejoría más lenta; además, se asocian con estancias hospitalarias prolongadas y con uso frecuente de antimicrobianos -incluso de amplio espectro-, y pueden llegar a ser fatales u ocasionar secuelas que deterioren la calidad de vida de quienes las padecen ^6^.

Algunos pacientes con errores innatos de la inmunidad pueden desarrollar neoplasias, fenómenos de desregulación inmunológica (autoinmunidad o autoinflamación) y enfermedades alérgicas ^5^.

Los errores innatos de la inmunidad se clasifican en 10 grupos, siendo los más frecuentes las inmunodeficiencias humorales (50-60 %), seguidas por las inmunodeficiencias combinadas (20-25 %), los defectos en la fagocitosis (5-10 %) y los defectos del complemento (5 %) ^6^. Estas estadísticas concuerdan con estudios nacionales como el realizado por Pedraza *et al*. en un hospital de cuarto nivel en Bogotá, con una población de 76 pacientes, en la que prevalecían las inmunodeficiencias humorales como primera causa (56 %), seguidas de las inmunodeficiencias combinadas (42 %) y los defectos en la fagocitosis (2,6 %) ^7^.

Por lo anterior, es primordial la prevención de las enfermedades infecciosas que podrían amenazar la vida en este grupo de riesgo a lo largo de su ciclo vital mediante la inmunoprevención, priorizando el uso de herramientas como la vacunación o los anticuerpos monoclonales ^1^.

La evolución de los pacientes con errores innatos de la inmunidad es muy heterogénea en cuanto a la propensión a infecciones y a las inmunizaciones. Se evidencia que la seguridad, eficacia, indicaciones y contraindicaciones de la vacunación, dependen del tipo y grado de afectación inmunológica, así como del tipo de vacuna ^1^.

Considerando lo expuesto, el propósito de esta búsqueda no sistemática fue proporcionar recomendaciones claras, fundamentadas en la evidencia clínica disponible, sobre la vacunación en la población pediátrica con los errores innatos de la inmunidad más prevalentes a nivel global. Se tomó en cuenta lo establecido en el Plan Ampliado de Inmunización colombiano para evitar retrasos en los esquemas de vacunación y garantizar la cobertura de poblaciones vulnerables que podrían beneficiarse de la inmunización. Además, se incluyó un selecto grupo de vacunas no incluidas en el PAI que representan una ayuda importante para este grupo de pacientes.

Se llevó a cabo una revisión narrativa, no sistemática, de artículos indexados en español y en inglés, con los términos: “errores innatos de la inmunidad”, “inmunodeficiencias primarias”, “vacunación en errores innatos de la inmunidad”, “tipos de vacunas”, “mecanismo de acción de las vacunas” y “vacunas vivas en errores innatos de la inmunidad”.

Para incluir recomendaciones basadas en datos estadísticos y consensos actualizados, se emplearon motores de búsqueda, como PubMed, Medline y ScienceDirect, y páginas web de instituciones reconocidas, como los *Centers for Disease Control and Prevention* (CDC).

## Tipos de vacunas

Existen múltiples clasificaciones para las vacunas, según su composición, síntesis o naturaleza ^8^. Es importante conocer los distintos tipos de vacuna porque cada uno induce una respuesta inmunológica diferente. El comprender su composición y mecanismo de acción, sus indicaciones y contraindicaciones permite determinar cuál es la más apropiada para los diferentes grupos poblacionales ^9^^-^^11^. Para efectos de esta revisión, las vacunas se clasificaron en vivas atenuadas e inactivadas.

### Vacunas vivas atenuadas

Las vacunas vivas atenuadas contienen agentes patógenos que se encuentran debilitados o alterados, seleccionados por ser menos virulentos que en su forma natural, tras sucesivos pases del microorganismo en medios de cultivo o por métodos genéticos ^2^^-^^9^; en su forma atenuada, el microorganismo genera una respuesta inmunitaria similar a la que produciría la infección natural en el huésped ^9^.

Cabe destacar que la seguridad de las vacunas atenuadas varía según la gravedad del error innato de la inmunidad; por ejemplo, en el caso de las inmunodeficiencias combinadas, puede ocurrir que la vacunación produzca infecciones diseminadas e, incluso, la muerte ^2^^,^^8^^,^^10^^-^^12^.

Las vacunas vivas atenuadas pueden ser bacterianas o virales: bacterianas, como la basada en el bacilo de Calmette-Guérin (BCG) contra la tuberculosis o la de la fiebre tifoidea oral; y virales, como aquellas contra la poliomielitis, la varicela, la fiebre amarilla, el rotavirus, el sarampión, la rubéola y la parotiditis.

En algunos estudios se han reportado infecciones inducidas por vacunas atenuadas en pacientes con diferentes errores innatos de la inmunidad, como la vacuna oral contra la poliomielitis ^13^^,^^14^, o contra el rotavirus ^15^, el bacilo de Calmette-Guérin ^16^, la varicela o el sarampión ^17^. Por lo anterior, es importante el cribado neonatal para detectar inmunodeficiencias combinadas graves ya que, en algunos países (como Colombia), se administra la vacuna BCG antes de autorizar el egreso del hospital del recién nacido, por lo cual se corre el riesgo de inmunizar inadvertidamente neonatos con alteraciones inmunológicas ^10^^,^^11^.

### Vacunas inactivadas

Las vacunas inactivadas pueden contener el microorganismo completo inactivado, fragmentado o modificado (mediante métodos físicos o químicos), o estar conformadas por subunidades de este, como polisacáridos, polisacáridos conjugados, toxoides o partículas viroides (*virus-like particles*, VLP) ^2^^,^^8^^,^^10^. Son menos inmunogénicas y tienen una vida media más corta que la de las vacunas vivas atenuadas; por todo lo anterior, no tienen la capacidad de generar la enfermedad o reactivarla, lo que hace que su uso sea seguro en casos de inmunodeficiencia ^2^^,^^9^^,^^10^.

Las vacunas inactivadas pueden ser bacterianas o virales. Algunas de las vacunas inactivadas bacterianas son aquellas contra tos ferina, *Haemophilus influenzae* de tipo B o meningococo B, y la vacuna meningocócica ACWY; además, contra neumococo 13-valente/PCV13 (disponible en Colombia dentro del PAI) y 23-valente/PPSV23, cólera (oral), fiebre tifoidea (parenteral), difteria y tétanos. Entre las inactivadas virales se encuentran aquellas dirigidas contra la poliomielitis (VIP, inyectable), la hepatitis A (HAV), la rabia, la influenza, la hepatitis B (HBV) y el papiloma humano (HPV).

Las vacunas inactivadas generalmente se consideran seguras en pacientes inmunocomprometidos, pero su inmunogenicidad (reacción inmunitaria) puede variar según el grado de compromiso y, en ocasiones, pueden llegar a ser innecesarias, especialmente en aquellos que reciben tratamiento con inmunoglobulina G (IgG) ^4^^,^^12^. Se debe considerar su administración únicamente cuando se considere que puede generar un beneficio ^12^.

En el cuadro 1, se enumeran las vacunas vivas atenuadas e inactivadas que se contemplan en el plan ampliado de inmunizaciones en Colombia.

**Cuadro 1.** Plan ampliado de inmunización colombiano

Edad	Vacuna	Dosis
Recién nacido	BCG*	Única
	Hepatitis B	Recién nacido
2 meses	Pentavalente	Primera
	Poliomielitis	Primera
	Rotavirus	Primera
	Neumococo (PCV13)	Primera
4 meses	Pentavalente	Segunda
	Poliomielitis	Segunda
	Rotavirus	Segunda
	Neumococo (PCV13)	Segunda
6 meses	Pentavalente	Tercera
	Poliomielitis	Tercera
	Influenza estacional	Primera
7 meses	Influenza estacional	Segunda
12 meses	SRP*	Primera
	Varicela*	Primera
	Neumococo (PCV13)	Tercera
	Hepatitis A	Única
18 meses	Pentavalente	Primer refuerzo
	Poliomielitis	Primer refuerzo
	Fiebre amarilla*	Única
	SRP*	Refuerzo
5 años	DPT	Segundo refuerzo
	Poliomielitis	Segundo refuerzo
	Varicela*	Refuerzo
Niños de 9 años	Virus del papiloma humano (HPV)	Dosis única
Niñas de 9 a 17 años	Virus del papiloma humano (HPV)	Dosis única

BCG: bacilo de Calmette-Guérin; SRP: sarampión, rubeola, paperas; DPT: difteria, tétanos y pertussis

* Vacunas vivas atenuadas

## Recomendaciones para errores innatos específicos de la inmunidad

### Inmunodeficiencias humorales

Son alteraciones de la inmunidad que afectan principalmente la función de las células B y comprometen la normal reacción de los anticuerpos ^18^. La mayoría de las personas que las padecen no produce anticuerpos específicos ante exposiciones antigénicas, ya sea en forma natural o mediante inmunización activa, y en los casos más graves, se requiere tratamiento con IgG (^12^^,^^18^^,^^19^. Las indicaciones respecto a la vacunación dependen de la gravedad de dichas alteraciones, sean leves o graves.

*Leves*. Entre estas inmunodeficiencias humorales, se encuentran las deficiencias sintomáticas de las subclases IgAo IgG, y la deficiencia selectiva de anticuerpos específicos con concentraciones normales de inmunoglobulinas ^12^.

La evidencia clínica sugiere que estos pacientes pueden ser vacunados de manera segura con vacunas inactivadas o con las vivas atenuadas ^12^^,^^18^. Aunque la reacción humoral frente a la vacunación en estos individuos puede estar disminuida, usualmente se logra alcanzar una reacción protectora ^12^. Se cuenta con algunas excepciones, como con la VOP, ya que, según múltiples revisiones de casos, la administración de esta vacuna se ha asociado con el desarrollo de poliomielitis paralítica, especialmente en pacientes con inmunodeficiencias humorales ^1^^,^^19^^-^^21^. Por lo tanto, esta vacuna se considera contraindicada en pacientes que padezcan estas deficiencias.

*Graves.* Entre estas inmunodeficiencias humorales, se encuentran la inmunodeficiencia variable común y la agammaglobulinemia ligada al cromosoma X. En estas inmunodeficiencias graves, está contraindicada la administración de vacunas vivas atenuadas (como la triple viral -SRP-, la BCG, contra la fiebre amarilla o contra la varicela), debido a que estos pacientes presentan un mayor daño en su reacción humoral, el cual requiere usualmente tratamiento con IgG ^5^^,^^13^. En ellos existe un gran riesgo de desarrollar enfermedad diseminada, asociada con una respuesta deficiente de los anticuerpos o con la neutralización de la vacuna asociada con el uso terapéutico de la IgG ^4^^,^^12^^,^^18^.

En la mayoría de estos pacientes, se encuentra que las vacunas inactivadas tampoco resultan efectivas o necesarias debido al uso de IgG ^4^^,^^12^, con excepción de las vacunas contra la influenza, la hepatitis A, el papiloma humano, el meningococo y el SARS-CoV-2. Esto se debe a que, usualmente, las preparaciones de inmunoglobulina no contienen anticuerpos contra las cepas circulantes y la vacuna podría inducir una beneficiosa inmunidad celular ^19^^,^^20^^,^^22^. Algo similar ocurre con la administración de anticuerpos monoclonales, como palivizumab; por esto, se recomienda su aplicación, incluso, en pacientes que se encuentren recibiendo inmunoterapia intravenosa ^12^.

En cuanto a la vacunación contra el meningococo, hay dos clases principales de vacunas utilizadas contra *Neisseria meningitidis.* Las vacunas de polisacáridos están compuestas de polisacáridos bacterianos de la pared celular, purificados, mientras que las vacunas conjugadas se fabrican mediante la unión covalente de un antígeno a una proteína transportadora inmunogénica (por ejemplo, toxoide tetánico, toxoide diftérico o variante toxoide diftérico [CRM197]) para mejorar y mantener la célula B activa. Las vacunas deben escogerse según los serogrupos circulantes más prevalentes o causantes de un brote específico ^12^^,^^18^. En el cuadro 2 se encuentran las vacunas meningocócicas conjugadas disponibles en Colombia contra los serogrupos A, C, W y Y ^23^^,^^24^.

**Cuadro 2:** Vacunas meningocócicas conjugadas contra los serogrupos CWY disponibles en Colombia y recomendaciones asociadas con cada una de ellas

	Nimenrix (TT) (Pfizer)	Menveo (CRM197) (GSK)	Menactra (DT) (Sanofi)
Edad mínima	6 semanas	2 meses	9 meses
Edad máxima	> 55 años	55 años	55 años
Esquema	2+1 < 6 meses 1+1 = 6-12 meses 1 dosis > 12 meses	2+1 < 6 meses 2 dosis = 7-23 meses 1 dosis > 2 años	2 dosis = 9-23 meses 1 dosis > 2-55 años Refuerzo = 15-56 años
Persistencia	~ 10 años	~ 5 años	~ 5 años
Coadministración	PCV-10, PCV-13, PCV-15, PCV-20, SRP, SRPV, VHA, Hexavalente, HPV2	PCV-7, PCV-13, PCV- 15, PCV-20, FT, VHA, hexavalente, SRP, FA, SRPV, varicela, EJ, rabia, 4CMenB MenB-4C; DTPa; HPV41	DTPa, VHA, SRP, SRPV, varicela, FT No PCV-13

PCV 7, PCV10, PCV13, PCV15, PCV 20: vacuna neumocócica conjugada contra 7, 10, 13, 15 y 20 serotipos; SRP: triple viral (sarampión, rubeola y parotiditis); SRPV: vacuna contra sarampión, rubeola, parotiditis y varicela; VHA: vacuna contra hepatitis A; HPV2 y HPV41: vacuna contra el virus del papiloma humano de subtipos 2 y 41; FT: fiebre tifoidea; FA: fiebre amarilla; EJ: encefalitis japonesa; DTPa: vacuna contra tétanos, difteria y tos ferina acelular

Se sugiere que en la vacunación antineumocócica se utilice siempre un esquema terapéutico combinado secuencial de la PCV13 3 + 1 (tres dosis iniciales y una de refuerzo) y, posteriormente, con un intervalo mínimo de 8 semanas, una dosis de PSPV23. Esto se debe a que la PSPV23 es una vacuna independiente del timo que solo se administra a los mayores de dos años, por lo cual podría esperarse una disminución de la inmunidad a largo plazo. Sin embargo, los estudios demuestran que la administración previa de PCV13 puede actuar como *primer*, permitiendo que la PSPV23 permita una reacción inmunológica mejor y más prolongada contra los serogrupos no incluidos en la vacuna conjugada ^1^^,^^12^^,^^18^.

La administración de las demás vacunas inactivadas lo determina el especialista en inmunología o infectología según su criterio clínico, de acuerdo con las recomendaciones de los CDC, independientemente de que el paciente se encuentre recibiendo IgG ^12^^,^^18^.

**Figura 1.**
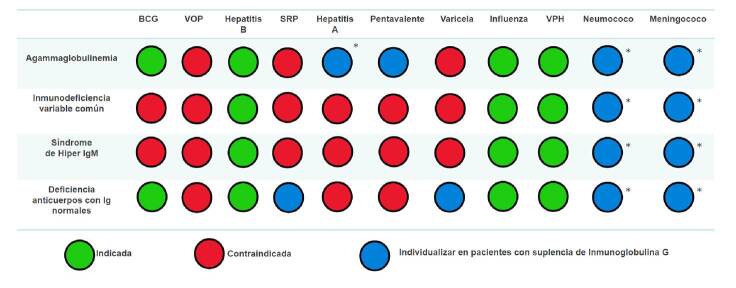
Indicaciones de vacunación del Programa Ampliado de Inmunizaciones y vacunas especiales en inmunodeficiencias humorales que reciben tratamiento sustitutivo con inmunoglobulina G intravenosa o subcutánea * Vacunas inactivadas cuya administración debe considerarse en algunos casos según el criterio clínico de los especialistas en inmunología e infectología, independientemente de la administración de inmunoglobulina G

Las recomendaciones de inmunización en pacientes con inmunodeficiencias humorales se resumen en las figuras 1 y 2. Las vacunas recomendadas durante la administración de inmunoglobulina G intravenosa o subcutánea, son aquellas contra influenza, SARS-CoV-2 y anticuerpos monoclonales específicos (palivizumab *No PAI*).

**Figura 2.**
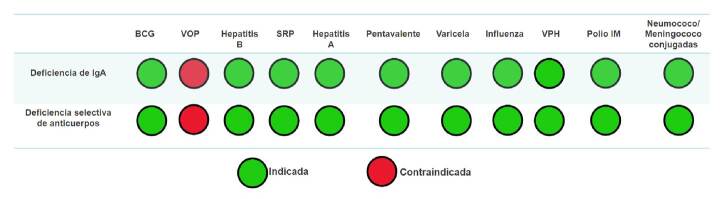
Indicaciones de vacunación del Programa Ampliado de Inmunizaciones y vacunas especiales en pacientes con inmunodeficiencias humorales que no reciben tratamiento sustitutivo con inmunoglobulina G intravenosa o subcutánea

### Inmunodeficiencias combinadas

Son alteraciones de la inmunidad que afectan principalmente la función de las células T de manera individual o combinada y, también, otros componentes del sistema inmunológico. Esto genera un compromiso variable de la inmunidad celular y la humoral y, por lo tanto, las recomendaciones de inmunización varían según el tipo y el grado de afectación ^12^^,^^18^, como se explica a continuación.

Respecto a la inmunización activa, en casos de inmunodeficiencias combinadas, usualmente se contraindica el uso de vacunas vivas por el riesgo de causar reacciones adversas graves, e incluso fatales, asociadas con el desarrollo de la enfermedad por diversas cepas vacunales. Su indicación se debe individualizar según el grado de afectación de la inmunidad celular ^18^. Por lo general, estos pacientes requieren administración de IgG, por lo cual las vacunas inactivadas no se encuentran contraindicadas, aunque su administración puede resultar fútil. No obstante, se recomienda aplicar las vacunas contra la influenza y contra el SARS-CoV-2, y administrar anticuerpos monoclonales específicos, como el palivizumab ^12^^,^^18^.

*Leves*. A este tipo de inmunodeficiencias combinadas pertenecen la ataxia telangiectasia, el síndrome incompleto de microdeleción 22q11.2 y el síndrome de Wiskott-Aldrich.

Constituyen un grupo de enfermedades heterogéneas caracterizadas por manifestarse con diversos grados de disfunción inmunológica celular y humoral ^18^. A pesar de que, en estas inmunodeficiencias leves, la eficacia de las vacunas inactivadas podría ser subóptima o nula, se considera que los pacientes que tengan una reacción residual de anticuerpos y que no se encuentren recibiendo IgG podrían beneficiarse de la administración de vacunas inactivadas ^12^^,^^18^^,^^20^. En el caso de aquellos que requieran de tratamiento con IgG, se debe suspender o evitar la administración de las vacunas inactivadas establecidas en el PAI, con excepción de las vacunas contra la hepatitis B, la influenza y el SARS-CoV-2. Además, se recomienda administrar anticuerpos monoclonales específicos contra el virus sincitial respiratorio (SRV), como el palivizumab. Las demás vacunas inactivadas podrían administrarse según el criterio de los especialistas en inmunología o infectología ^12^^,^^18^.

Para garantizar la seguridad de la inmunización activa en este grupo de pacientes, se sugiere practicar estudios inmunológicos tres meses antes de aplicar vacunas vivas atenuadas, para evaluar el riesgo y el beneficio de manera individualizada ^18^. Según Bonilla ^1^, Galicchio ^18^ y Khalili ^19^, los criterios para establecer la pertinencia de administrar dichas vacunas en pacientes con inmunodeficiencia son: recuento de linfocitos T CD4+ mayor de 500 células/ml, recuento de linfocitos T CD8+ mayor de 200 células/ml y reacción linfocitaria proliferativa normal a los mitógenos.

Ataxia telangiectasia. Se caracteriza por neurodegeneración progresiva, inmunodeficiencia, anormalidades cutáneas y gran incidencia de enfermedades oncológicas. Su manejo es variable y depende del grado de compromiso inmunológico; en ocasiones, puede requerirse IgG intravenosa o profilaxis antibiótica ^1^^,^^12^^,^^18^. Todas las vacunas inactivadas se consideran seguras; no obstante, considerando que su eficacia puede llegar a ser nula o subóptima, se sugiere el estricto seguimiento de la seroconversion del paciente ^12^^,^^18^. Los pacientes que se encuentren en tratamiento con IgG no deben recibir las vacunas inactivadas establecidas en el PAI, con excepción de aquellas contra la hepatitis B y la influenza ^4^^,^^12^^,^^18^.

La mayoría de los enfermos con ataxia telangiectasia son vacunados con BCG al nacimiento (antes del diagnóstico) y, hasta el momento, no se han documentado complicaciones asociadas ^18^.

Síndrome incompleto de microdeleción 22q11.2. Comprende un grupo heterogéneo de pacientes que pueden presentar dimorfismo facial, cardiopatía congénita, anomalías velopalatinas y paratiroideas, con inmunodeficiencia predominantemente celular leve y transitoria o sin ella ^12^^,^^18^.

En múltiples estudios se han analizado la seguridad y los eventos adversos posteriores a la inmunización con vacunas vivas en pacientes con síndrome de microdeleción 22q11.2. Se destaca el estudio de cohorte, retrospectivo y multicéntrico de Hofstetter *et al*., quienes evaluaron las enfermedades prevenibles con vacunas vivas, la cobertura, la puntualidad y los eventos supuestamente atribuidos a la vacunación o inmunización durante los 56 días siguientes a la vacunación. Así, concluyeron que, en general, las vacunas vivas fueron bien toleradas por los pacientes con este síndrome y con inmunosupresión de leve a moderada ^18^^,^^25^^-^^27^.

Por otra parte, se considera que las vacunas inactivadas incluidas dentro del PAI se pueden administrar de manera segura según la edad; asimismo sucede con las vacunas contra la influenza, el neumococo y el meningococo, en aquellos pacientes que no se encuentren recibiendo IgG ^1^^,^^3^^,^^18^. En caso contrario, cuando el paciente recibe inmunoglobulina G, se deben respetar las mismas recomendaciones descritas anteriormente.

Síndrome de Wiskott-Aldrich. Es un trastorno de herencia recesiva ligado al cromosoma X que se caracteriza por eczema, trombocitopenia con microplaquetas y compromiso progresivo del sistema inmunológico, celular y humoral ^12^^,^^18^. En este síndrome se considera segura la administración de vacunas inactivadas; no obstante, su eficiencia podría ser subóptima, por lo que se sugiere seguimiento estricto de la seroconversion ^18^. En el caso de los pacientes cuyo tratamiento incluya IgG, se deben considerar las mismas recomendaciones descritas previamente ^1^^,^^4^^,^^11^^,^^18^.

**Figura 3.**
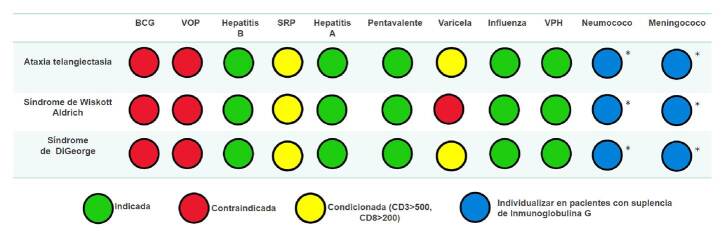
Indicaciones de vacunación del Programa Ampliado de Inmunizaciones y vacunas especiales en inmunodeficiencias combinadas leves. * Vacunasinactivadas cuya administración debe considerarse en algunos casos según el criterio clínico de especialistas en inmunología e infectología, independientemente de la administración de inmunoglobulina G

Las recomendaciones sobre la inmunización en pacientes con inmunodeficiencias combinadas leves se resumen en la figura 3.

*Graves.* A este tipo de inmunodeficiencias combinadas pertenecen la inmunodeficiencia combinada grave y el síndrome completo de microdeleción 22q11.2.

Constituyen un conjunto de síndromes de herencia autosómica recesiva ^18^ que se pueden dividir en típicos-linfopenia T acentuada, agammaglobulinemia y ausencia de la función inmunitaria celular y humoral- y atípicos, que comparten la deficiencia inmunitaria celular y humoral. Los pacientes requieren usualmente opciones terapéuticas como trasplante de progenitores hematopoyéticos o terapia génica ^12^^,^^18^.

En este grupo de pacientes con inmunodeficiencias combinadas graves, se encuentra absolutamente contraindicada la administración de vacunas vivas atenuadas ^12^^,^^18^ por su asociación con el desarrollo de enfermedad diseminada. Se han reportado múltiples casos de infección por rotavirus ^15^^,^^28^^,^^29^, poliomielitis y poliomielitis paralítica y excreción del virus vivo después de la vacunación con VOP ^30^^-^^33^, y tuberculosis diseminada posterior a la administración de BCG ^34^, en niños con inmunodeficiencias combinadas graves y en niños ^34^, complicaciones diseminadas asociadas con la BCG ^12^.

Marciano *et al*. realizaron un estudio con 349 pacientes con inmunodeficiencia combinada grave vacunados contra BCG, de los cuales, 46 fallecieron, 34 desarrollaron infección diseminada y, 171, infección localizada. Los autores concluyeron que la vacuna BCG tiene una alta tasa de complicaciones en pacientes con inmunodeficiencias combinadas graves, incrementando la mortalidad, y que su administración en pacientes con menos de 250 linfocitos T por mililitro es más riesgosa ^12^^,^^34^.

Además, en un estudio realizado por Pöyhönen *et al*., identificaron que en una muestra de 107 pacientes con poliomielitis paralítica posterior a la vacuna, la tercera parte padecía inmunodeficiencias combinadas leves y graves ^12^^,^^35^.

Por otra parte, se considera que las vacunas inactivadas no brindan mayor beneficio en las inmunodeficiencias combinadas graves ^12^, no solo porque no habrá una reacción inmunitaria adecuada, sino porque, normalmente, estos pacientes se encuentran en tratamiento con inmunoglobulina G ^1^^,^^4^^,^^12^^,^^18^.

La vacuna contra el virus del papiloma humano (HPV) puede ser de utilidad específicamente en alteraciones que incrementan la vulnerabilidad ante estos virus, como la inmunodeficiencia combinada grave, el síndrome de Wiskott-Aldrich y el síndrome de ataxia- telangiectasia. Independientemente de la edad, se recomienda el esquema de tres dosis de la vacuna nonavalente: segunda dosis después de uno a dos meses de la primera y la tercera dosis a los seis meses. Es importante destacar que, en la actualidad, esta vacuna no se encuentra dentro del PAI colombiano; sin embargo, se recomienda su aplicación para evitar infección grave o diseminada en este grupo de pacientes ^1^^,^^12^.

### Defectos de la fagocitosis

Los principales defectos de la fagocitosis comprenden la enfermedad granulomatosa crónica y las deficiencias de las moléculas de adhesión.

*Enfermedad granulomatosa crónica*. Es un trastorno hereditario en el cual hay una disfunción de la enzima NADPH oxidasa que impide a las células fagocíticas generar especies reactivas de oxígeno, lo que predispone a infecciones recurrentes graves ^1^^,^^18^^,^^19^. Estos pacientes son particularmente propensos a infecciones por micobacterias y bacterias con expresión de catalasas; se ha documentado que, en los países donde la tuberculosis continúa siendo endémica, son más frecuentes las infecciones por micobacterias en los pacientes con enfermedad granulomatosa crónica ^1^^,^^36^.

En un estudio se demostró que hasta el 75 % de los pacientes con esta enfermedad presentaba infección por BCG asociada con una mortalidad del 18 % y que, debido a la vacunación temprana, usualmente las infecciones por BCG suelen ser la primera manifestación clínica de los pacientes con este tipo de error inmunológico. Por lo anterior, se sugiere un tamizaje oportuno para evitar eventos adversos asociados con la vacunación ^36^^-^^38^.

Teniendo en cuenta lo anterior, en este grupo de pacientes se contraindica de manera absoluta la administración de vacunas bacterianas vivas, ya que se han reportado múltiples casos de infección diseminada por BCG asociada con la vacunación ^37^^,^^39^^-^^41^. Las vacunas virales atenuadas -como la triple viral y la vacuna de la varicela- no tienen ninguna contraindicación y pueden administrarse de manera segura en pacientes con enfermedad granulomatosa crónica ^19^^,^^20^. Se considera segura y eficaz la administración de vacunas inactivadas, pues se espera una adecuada respuesta de los anticuerpos ^1^^,^^18^.

En estos pacientes, entre las vacunas especiales, es importante administrar la de la influenza anual para disminuir la carga de morbimortalidad por la coinfección con *Staphylococcus aureus*, frecuente en estos casos, y también, la vacuna actualizada contra la COVID-19 ^12^^,^^20^.

*Deficiencias de moléculas de adhesión*. Este grupo de defectos se caracteriza por la falta de expresión de las moléculas de adhesión ^1^^,^^18^. Se manifiesta clínicamente por infecciones sin material purulento en las lesiones, retardo en la caída del cordón umbilical y leucocitosis con neutrofilia ^18^.

En estos pacientes se contraindica, de manera absoluta, la administración de vacunas de microorganismos vivos, tanto bacterianas como virales ^1^^,^^4^^,^^12^^,^^18^. Por otra parte, se considera que las vacunas inactivadas se pueden utilizar de manera segura y eficaz ^12^^,^^18^.

**Figura 4.**
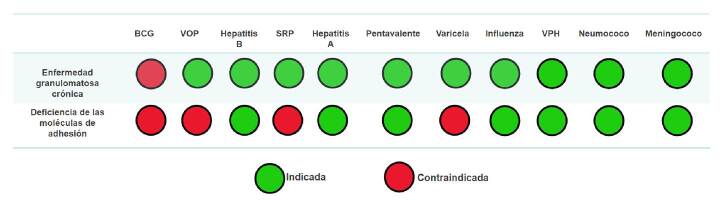
Indicaciones de vacunación del Programa Ampliado de Inmunizaciones y vacunas especiales en defectos de la fagocitosis

Es importante tener en cuenta que, en general, se recomienda la administración de vacunas especiales en los pacientes con defectos de la fagocitosis, como aquellas contra influenza, meningococo, neumococo y varicela; esta última se encuentra contraindicada en pacientes con deficiencia de las moléculas de adhesión ^1^^,^^12^^,^^18^. Las recomendaciones sobre inmunización en casos de defectos de la fagocitosis se muestran en la figura 4.

### Defectos del complemento

Existen deficiencias genéticas en todos los componentes del sistema del complemento; sin embargo, no hay alteración de la respuesta inmunológica celular o humoral en estos casos ^18^^-^^21^. Su compromiso clínico generalmente abarca infecciones bacterianas por gérmenes encapsulados y autoinmunidad ^12^^,^^18^.

**Figura 5.**
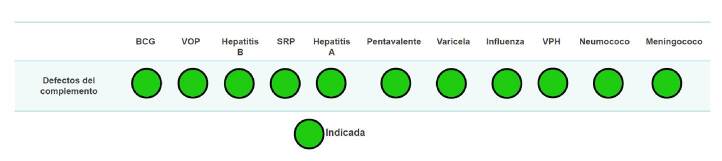
Indicaciones de vacunación del programa ampliado de inmunizaciones y vacunas especiales en defectos del complemento

Por lo anterior, se considera segura y efectiva la inmunización activa de estos pacientes con todas las vacunas incluidas en el PAI y con la vacuna de la influenza anual ^12^^,^^18^^-^^19^, y se recomienda fuertemente considerar la vacunación contra los organismos específicos a los cuales son más vulnerables (*H. influenzae* de tipo b, *Neisseria meningitidis, Streptococcus pneumoniae*), bien sea con vacunas conjugadas o con polisacáridas, según la edad ^18^^-^^20^^,^^42^^,^^43^. Las recomendaciones sobre inmunización en pacientes con defectos del complemento se muestran en la figura 5.

## Recomendaciones sobre vacunación contra el SARS-CoV-2

En múltiples estudios se ha demostrado que las personas que padecen de errores innatos de la inmunidad tienen un mayor riesgo de desarrollar formas graves de la COVID-19^44^^,^^45^, por lo cual en los últimos años se ha estado evaluando si una inmunización activa “agresiva” en estos pacientes podría resultar beneficiosa ^46^.

Las vacunas de ARN mensajero (ARNm) han demostrado ser seguras y efectivas para prevenir formas graves de la enfermedad y muertes, según hallazgos de más de 20 estudios sobre la inmunogenicidad a la vacuna contra SARS-CoV-2 en errores innatos de la inmunidad ^47^. Delmonte *et al*. publicaron una de las primeras cohortes de pacientes con diversos errores innatos inmunes, en la cual compararon la reacción inmunológica de 81 pacientes a un esquema de vacunas de mRNA; encontraron que el 85 % de ellos desarrolló anticuerpos anti-S, detectables, después de completar su esquema primario de vacunación ^48^. Por su parte, Hagin *et al*. evaluaron las respuestas humoral y celular frente a la vacuna contra SARS-CoV-2, en una cohorte de 16 pacientes con diferentes errores innatos; concluyeron que la vacunación era segura y que, en la mayoría de los casos, se producía una respuesta específica de anticuerpos ^46^.

A la fecha, las vacunas contra SARS-CoV-2 han demostrado un excelente perfil de seguridad en pacientes con errores innatos de la inmunidad y no existe evidencia de que tengan mayor riesgo de presentar efectos adversos después de la vacunación ^46^. Se han encontrado reportes de mayor inmunogenicidad en pacientes inmunocomprometidos después de recibir la dosis de refuerzo ^49^^,^^50^. Por esto, en las últimas recomendaciones de los CDC sobre vacunación en grupos especiales, se sugiere un esquema primario de tres dosis de vacuna de ARNm con una diferencia de 1 a 2 meses entre cada aplicación, según indique el fabricante; además, se recomienda uno o dos refuerzos, al menos, dos meses después de la última dosis, en pacientes con depleción de células B, trasplante de células madres o en tratamiento con linfocitos T con receptor quimérico para el antígeno, CAR-T (*chimeric antigen receptor-Tcell therapy*), en forma similar al esquema utilizado en población sana ^46^^-^^50^. En el cuadro 3 se muestra el esquema de vacunación contra SARS-CoV-2 usado en Colombia.

**Cuadro 3.** Esquema de vacunación contra SARS-CoV-2 en Colombia (2024)

Población	Indicaciones
≥ 6 meses	Esquema primario de dos dosis de vacuna de ARNm
> 12 años	Dosis adicional con vacuna de ARNm al menos seis meses después de la última dosis:
	- Personas de 12-17 años después de recibir el esquema primario
	- Mayores de18 años al transcurrir seis meses después de la última dosis
Grupos priorizados para dosis adicional	- Mujeres gestantes: una dosis por cada embarazo y debe ser de ARNm Pfizer*
	- Población con comorbilidades (hipertensión arterial, diabetes mellitus, enfermedad pulmonar obstructiva crónica -EPOC-, cáncer, enfermedad renal, inmunodeficiencias)
	- Mayores de 60 años
	- Talento humano en salud

Cabe esperar que las inmunodeficiencias y tratamientos que comprometan la reacción de las células B y, en consecuencia, la generación de anticuerpos, sean las que más comprometen la eficacia de la vacuna; sin embargo, no hay suficiente información disponible al respecto, ni sobre el papel de la inmunidad celular inducida con la vacunación en estos pacientes ^46^^-^^50^.

## Vacunas próximas por llegar a Colombia

*Vacuna recombinante contra meningococo del serogrupo B* (MenB-4C); se trata de una vacuna conjugada que contiene una proteína de unión al factor H. Esta vacuna MenB-4C se recomienda en pacientes con inmunodeficiencias asociadas con defectos del complemento, quienes se encuentran en mayor riesgo de infección por microorganismos encapsulados debido a las deficiencias en la opsonización, la activación de la anafilotoxina del complemento o la actividad bactericida sérica, mecanismos importantes para eliminar bacterias encapsuladas. El *Advisory Committee on Immunization Practices* (ACIP) avala el uso de la MenB-4C en pacientes mayores de 10 años con gran riesgo de enfermedad meningocócica, en un esquema de dos dosis con un intervalo de un mes ^51^^,^^52^.

*Vacuna contra el dengue AK-003* (QDENGA^®^): esta es una vacuna tetravalente, viva y atenuada, recomendada por la OMS para la población pediátrica en entornos de gran carga de dengue e importante intensidad de transmisión. Se sugiere un esquema de dos dosis con un intervalo de tres meses a partir de los cuatro años de vida. Hasta el momento no existen estudios en personas inmunocomprometidas, por lo cual su uso en ellas está contraindicado ^53^.

*Vacunas antineumocócicas conjugadas*: la 15-valente (PCV15 o Vaxneuvance^®^) y la 20-valente (PCV20 o Prevnar 20^®^) están aprobadas por la *Federal Drug Administration* (FDA) de los Estados Unidos para prevenir la enfermedad causada por la bacteria *S. pneumoniae*. La PCV15 está aprobada para niños y adultos, y la PCV20, solo para adultos ^54^.

## Conclusión

Los pacientes afectados por errores innatos de la inmunidad son más propensos a infecciones, por lo que la eficacia, la seguridad y la inmunogenicidad de las vacunas son factores para tener en cuenta en dicha población. En algunos de estos errores innatos, las vacunas de microorganismos vivos pueden resultar en enfermedad diseminada y se contraindican de manera absoluta. Por lo tanto, es importante evaluar de forma individualizada la capacidad de reacción de cada paciente.

En general, las vacunas de microorganismos muertos son seguras y se pueden aplicar en todos los pacientes con capacidad de generar una respuesta inmunitaria. En algunos errores innatos de la inmunidad se requieren vacunas especiales, adicionales a las que se encuentran en el PAI colombiano, como en los casos de defectos del complemento o asplenia.

El suplemento de IgG reduce la eficacia de la mayoría de las vacunas, por lo cual las inactivadas podrían llegar ser prescindibles. Sin embargo, cada paciente debe ser valorado individualmente, en conjunto con un especialista en inmunología o infectología.

Cada vez hay mayor evidencia clínica sobre la administración de productos biológicos específicos (como palivizumab) en la inmunoprevención de enfermedades específicas, como las infecciones por el virus sincitial respiratorio

La vacunación en pacientes con errores Innatos de la Inmunidad es segura y efectiva, y reduce la carga de morbimortalidad asociada con infecciones recurrentes. No debe retrasarse el calendario vacunal mientras el paciente tenga una adecuada capacidad de reacción inmunológica.
